# Corrosion Inhibition of Cast Iron in Arabian Gulf Seawater by Two Different Ionic Liquids

**DOI:** 10.3390/ma8073883

**Published:** 2015-06-26

**Authors:** El-Sayed M. Sherif, Hany S. Abdo, Sherif Zein El Abedin

**Affiliations:** 1 Deanship of Scientific Research (DSR), Center of Excellence for Research in Engineering Materials (CEREM), Advanced Manufacturing Institute (AMI), King Saud University, P.O. Box 800, Al-Riyadh 11421, Saudi Arabia; E-Mail: habdo@ksu.edu.sa; 2 Electrochemistry and Corrosion Laboratory, Physical Chemistry Department, National Research Centre, El Bohouth St. 33, Dokki, Giza 12622, Egypt; 3 Mechanical Design and Materials Department, Faculty of Energy Engineering, Aswan University, Aswan 81521, Egypt; 4 Institute of Electrochemistry, Clausthal University of Technology, Arnold-Sommerfeld-Str. 6, Clausthal-Zellerfeld D-38678, Germany

**Keywords:** corrosion, inhibitors, ionic liquids, cast iron, seawater, weight-loss

## Abstract

In this paper we report on the corrosion inhibition of cast iron in Arabian Gulf seawater by two different ionic liquids namely, 1-ethyl-3-methylimidazolium chloride ([EMIm]Cl) and 1-butyl-1-methylpyrrolidinium chloride ([Py_1,4_]Cl). The inhibiting influence of the employed ionic liquids was investigated by weight loss, open circuit potential electrochemical impedance spectroscopy, and cyclic potentiodynamic polarization. The results show the corrosion inhibition impact of the employed ionic liquids (ILs). Compared with [Py_1,4_]Cl, [EMIm]Cl shows a higher inhibition efficiency at a short immersion time, for the examined ILs concentrations. However, [Py_1,4_]Cl exhibits a higher efficiency upon increasing the immersion time indicating the persistence of the inhibiting influence. The corrosion inhibition of the employed ionic liquids is attributed to the adsorption of the cations of the ionic liquids onto the surface of cast iron forming a corrosion barrier.

## 1. Introduction

Iron and its alloys are widely employed as construction materials owing to their distinctive mechanical properties and low cost. They are utilized in many industries, in a direct contact with corrosive environments, as, e.g., tanks, pipes, boilers, oil and gas production units, refineries, *etc.* Therefore, corrosion inhibition regimes are routinely applied in order to control the corrosion attack. One of the most effective and economic approaches to mitigate corrosion is the use of organic inhibitors. The inhibition is usually achieved by adsorption of organic molecules onto the metallic surface forming a barrier between the surface and the corrosive environment. The adsorptive interaction with the metal surface takes place through heteroatoms such as phosphorus, sulphur, nitrogen and oxygen as well as through triple bonds or aromatic rings blocking the active corrosion sites [1,2,3,4]. The environmental impact of many organic inhibitors has motivated researchers to find environmentally friendlier alternatives.

Currently, there is an increased interest in the application of ionic liquids (ILs) as corrosion inhibitors [5,6,7,8,9,10,11]. In general, ionic liquids are salts with melting temperatures below 100 °C. They are composed of large asymmetric organic cations such as, e.g., imidazolium, pyrrolidinium, pyridinium, phosphonium, and inorganic or organic anions such as, e.g., halide, sulfate, nitrate, dicyanamide, trifluoromethylsulfonate, bis(trifluoromethylsulfonyl) amide. Ionic liquids have exceptional physical properties making them attractive materials for a wide variety of applications [12,13,14,15,16,17,18]. In addition to their high thermal and electrochemical stabilities, ionic liquids are usually nonvolatile, nonflammable, and less toxic than conventional organic solvents. Therefore, they are regarded as an excellent alternative to replace volatile, environmentally hazardous organic solvents. The bulky structure of ionic liquids and the presence of heteroatoms that have lone electron pairs such as, N, S, O or P, make ionic liquids attractive candidates for corrosion inhibition. A large variety of potential cations and anions can be utilized for designing ionic liquids with preselected properties; more than one million simple ionic liquids can be obtained [17]. Hence, a broad range of ionic liquids can be employed as potential corrosion inhibitors. However, one of the principle challenges in the widespread applications of ionic liquids is the cost. To date, the cost of ionic liquids is a bit high, however, in our opinion; the cost should be weighed against the advantages of ionic liquids. A number of ionic liquids were reported to be efficient inhibitors for the corrosion of mild steel [5,6,7,8,9,10,11], aluminium [19,20,21] and copper [22] in acidic solutions. It was reported that the ionic liquids 1-ethyl-3-methylimidazolium dicyanamide [6], some selected vinylimidazolium bromide [7] and 1,3-dioctadecylimidazolium bromide and *N*-octadecylpyridinium bromide [9], are efficient inhibitors for the corrosion of mild steel in 1 M H_2_SO_4_ solutions. The inhibition impact of some bis(trifluoromethysulfonyl) amide imidazolium based ionic liquids [23], 1-butyl-3-methylimidazolium chloride [10], and 1-butyl-3-methylimidazolium bromide [11], on the corrosion of mild steel in 1 M HCl solutions was demonstrated. Quite recently, we showed the inhibiting effect of the ionic liquid 1-butyl-1-methylpyrrolidinium trifluoromethylsulfonate ([Py_1,4_]TfO) on the corrosion of mild steel in 3.5% NaCl solutions [24].

**Scheme 1:**
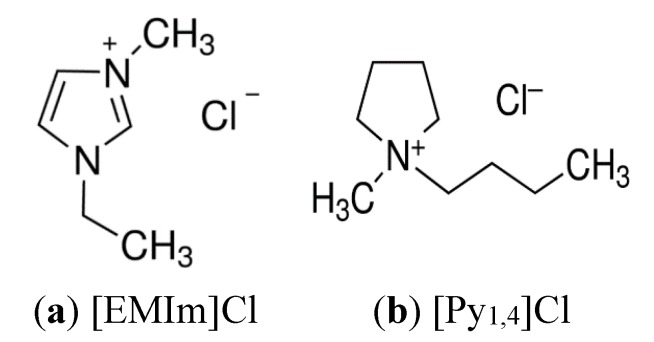
Chemical structure of (**a**) 1-ethyl-3-methylimidazolium chloride ([EMIm]Cl) and (**b**) 1-butyl-1-methylpyrrolidinium chloride ([Py_1,4_]Cl).

In the present paper, we have investigated the inhibiting influence of two different ionic liquids namely, 1-ethyl-3-methylimidazolium chloride ([EMIm]Cl) and 1-butyl-1-methylpyrrolidinium chloride ([Py_1,4_]Cl), on the corrosion of cast iron in Arabian Gulf seawater (AG-seawater). The chemical structure of (a) 1-ethyl-3-methylimidazolium chloride ([EMIm]Cl) and (b) 1-butyl-1-methylpyrrolidinium chloride ([Py_1,4_]Cl) are shown respectively in Scheme 1. Atkin *et al.*, [25] showed by atomic force microscopy (AFM) and scanning tunneling microscopy (STM) that the adsorptive interaction of pyrrolidinium-based ionic liquids with metallic surfaces is much stronger than that of imidazolium ones. Therefore, it seemed of interest to explore and to compare the corrosion inhibition influences of both [EMIm]Cl and [Py_1,4_]Cl.

## 2. Results and Discussion

### 2.1. Weight Loss Measurements

**Figure 1:**
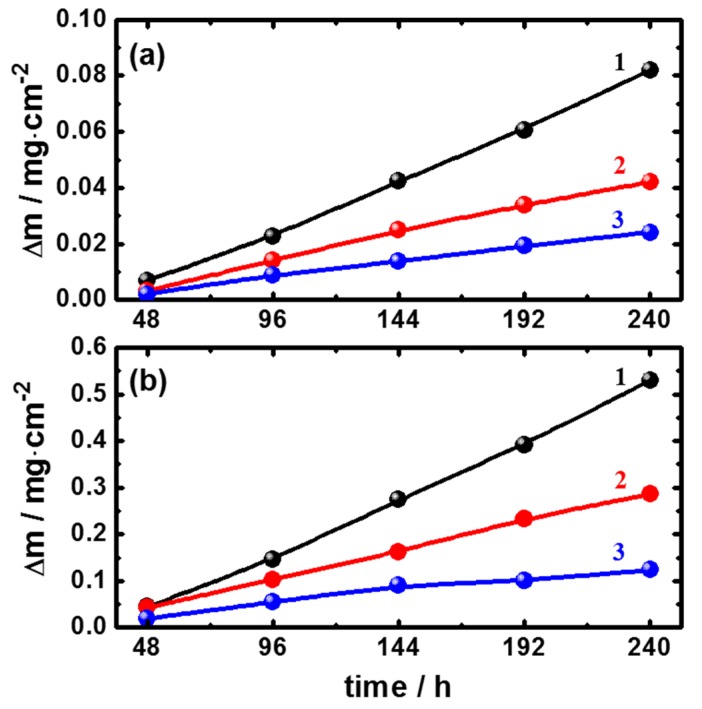
Change of the dissolution rate (Δm, mg·cm^2^) with time for the cast iron coupons immersed in Arabian Gulf (AG) seawater solutions without (1) and with (2) 1.0 mM and (3) 5.0 mM of (**a**) [EMIm]Cl and (**b**) [Py_1,4_]Cl, respectively.

In order to have an insight into the inhibiting effect of the employed ionic liquids, [EMIm]Cl and [Py_1,4_]Cl, on the corrosion of cast iron in AG-seawater, weight loss measurements for prolonged times, up to 10 days, were investigated. The weight loss of cast iron coupons in AG-seawater fee and containing two different concentrations, 1 and 5 mM, of the employed ionic liquids was determined after different immersion intervals to explore the influence of immersion time on the efficiency of the inhibitors. Figure 1 shows the variation of the weight loss of the cast iron samples in the uninhibited and inhibited solutions as a function of immersion time. As seen, the weight loss of the samples in the uninhibited solutions steadily increases with the increase in the immersion time signifying the increase in the corrosion rate.

In the presence of the employed ionic liquids, the weight loss is significantly reduced and the extent of the decrease in the weight loss is obvious as the immersion time increases. The decrease in the weight loss indicates the inhibiting influence of the employed ionic liquids. The inhibition effect can be attributed to the adsorption of the cations of the ionic liquids, either [EMIm]^+^ or [Py_1,4_]^+^, on the surface forming protective films against corrosion attack. For both ionic liquids, the inhibiting influence increases upon increasing the concentration as more cations can be adsorbed on the surface resulting in the increase in the surface coverage by the ionic liquids’ cations. As shown in Figure 1, at a given immersion time the observed weight loss decreases as the concentration increases. It should be noted that higher concentrations of the employed ionic liquids more than 5 mM did not show a further improvement in the performance of the in the inhibitors (the results are not shown). As known, once a protective layer is applied to the surface a further increase in the inhibitor concentration does not lead to enhanced inhibition.

**Figure 2:**
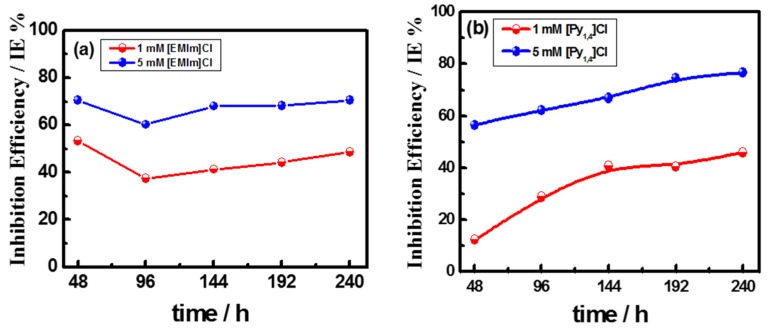
Inhibition efficiency of (**a**) [EMIm]Cl and (**b**) [Py_1,4_]Cl as a function of immersion time for cast iron coupons in AG-seawater solutions.

Figure 2 shows the variation of the inhibition efficiency of the employed ionic liquids, estimated from the weight loss data, as a function of immersion times. In the case of [EMIm]Cl, Figure 2a, the inhibition efficiencies estimated after 48 h immersion were 53% and 70% at concentrations of 1 mM and 5 mM, respectively. After 96 h immersion time, the inhibition efficiency showed a significant decrease then it slightly increased with the increase in the immersion time. This behavior might be attributable to desorption/adsorption of [EMIm]^+^ from/on the electrode surface. The decrease in the inhibition efficiency with the immersion time usually signifies the tendency of the inhibitor to be desorbed from the electrode surface. In the case of [Py_1,4_]Cl, Figure 2b, the inhibition efficiency rises with the immersion time signifying the stability of the adsorbed film. This can be indicative of the higher strength of adsorption of [Py_1,4_]^+^ compared to that of [EMIm]^+^. Compared with [Py_1,4_]Cl, [EMIm]Cl shows a higher inhibition efficiency at low immersion time, for the examined ILs concentrations. This might be attributable to the easier adsorption of [EMIm]^+^ due to the presence of more adsorption centres in the imidazoline ring compared with the pyrrolidine one. In case of [EMIm]Cl the adsorption might occur through the free electron pair on one nitrogen atom, π electrons, or electrostatic attraction between the charged [EMIm]^+^ and the metal surface. In [Py_1,4_]Cl, the adsorption can take place via electrostatic interaction of [Py_1,4_]^+^ with the surface. Therefore, higher inhibition is achieved after short term immersion in case of [EMIm]Cl. However, the strength of adsorption in the case of pyrrolidinium is higher and hence the inhibition is more persistent. As the charge on the [Py_1,4_]^+^ cation is localized whereas it is delocalized on the [EMIm]^+^ cation, the electrostatic interaction of [Py_1,4_]^+^ with the metal surface is stronger than that of [EMIm]^+^. Atkin *et al.* [25] showed by using atomic force microscopy that the strength of adsorption of [Py_1,4_]^+^ is about four times higher than that of [EMIm]^+^. This might account for the observed increase in the inhibition efficiency of [Py_1,4_]Cl upon increasing the immersion time.

The inhibition process of the corrosion of cast iron in AG-seawater by the employed ionic liquids can be described as the following: First, Cl^−^ is adsorbed on the electrode surface forming (FeCl^−^)_ads_, and by ongoing time the surface concentration of Cl^−^ increases leading to corrosion according to the following equation:

(FeCl^−^)_ads_ + Cl^−^ = FeCl_2_ + 2e^−^ (1)

In the inhibited electrolyte either [EMIm]^+^ or [Py_1,4_]^+^ cations can be adsorbed onto the electrode surface and electrostatically interact with (FeCl^−^)_ads_ species forming a protective layer:

(FeCl^−^)_ads_ + [Cation]^+^ = (FeCl^−^[Cation]^+^)_ads_ (2)

### 2.2. Open-Circuit Potential

**Figure 3:**
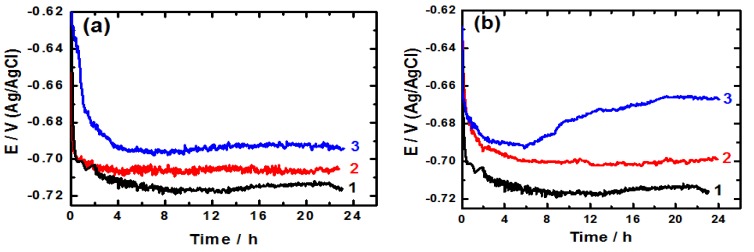
Change of the open-circuit potential with time for the cast iron electrodes immersed in AG-seawater solutions without (1) and with (2) 1.0 mM and (3) 5.0 mM of (**a**) [EMIm]Cl and (**b**) [Py_1,4_]Cl, respectively.

The influence of the employed ionic liquid inhibitors on the free corrosion potential (open-circuit potential) of the cast iron in AG-seawater was investigated. The variation of the open-circuit potential (OCP) of the electrode with time can give valuable information on the inhibition process. Figure 3 shows the change of the OCP of cast iron with time, for about 24 h, in AG-seawater free and containing two different concentrations of [EMIm]Cl (Figure 3a) and of [Py_1,4_]Cl (Figure 3b). As seen, the absence of ionic liquids the potential moves rapidly at the early moments of immersion towards the negative direction indicating the dissolution of the air-formed, pre-immersion oxide layer. Then the potential slightly shifts to more negative values with time as a result of the corrosion attack to the electrode surface. A quasi-steady state potential is attained at about 8 h of immersion due to the formation of a layer of the corrosion products on the electrode surface that can exert a sort of passivity to the electrode. On addition of [EMIm]Cl (Figure 3a), the same general feature is obtained, however the free corrosion potential shifts to less negative values and the potential shift rises as the concentration increases. This indicates the inhibiting influence of [EMIm]Cl on the corrosion of cast iron. In the case of [Py_1,4_]Cl (Figure 3b), the shift of the free corrosion potential towards less negative values is higher than that observed for [EMIm]Cl signifying the increase in the inhibiting influence of [Py_1,4_]Cl.

### 2.3. Electrochemical Impedance Spectroscopy Investigations

**Figure 4:**
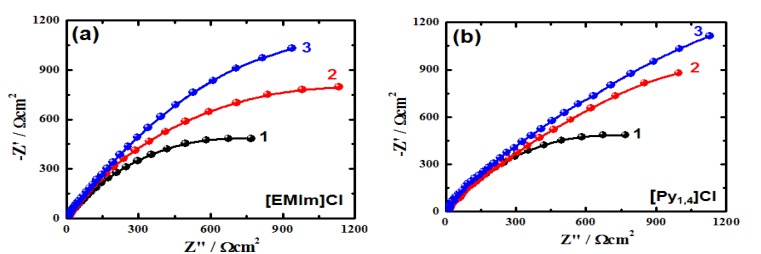
Typical Nyquist plots for the cast iron in AG-seawater without (1) and with (2) 1.0 mM and (3) 5.0 mM of [EMIm]Cl (**a**) and [Py_1,4_]Cl (**b**).

**Figure 5:**
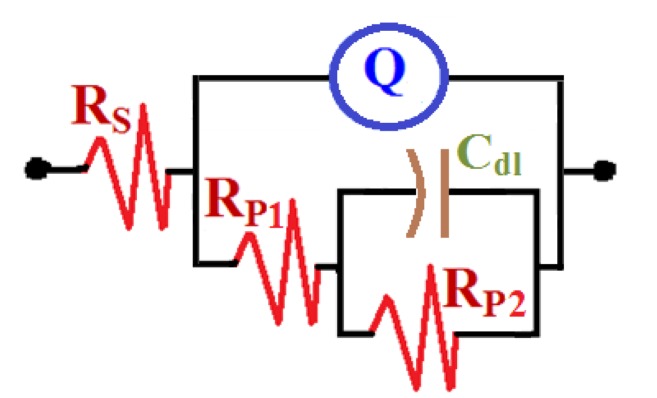
Equivalent circuit model used to fit the electrochemical impedance spectroscopy (EIS) data presented in Figure 4.

Electrochemical impedance spectroscopy (EIS) was also utilized to evaluate the inhibiting impact of the employed ionic liquids on the corrosion of cast iron in AG-seawater. This was attained by determining the kinetic parameters for electron transfer reactions at the iron/electrolyte interface from the Nyquist plots. Figure 4 displays the Nyquist plots of the cast iron in AG-seawater without and with addition of different concentrations of [EMIm]Cl and of [Py_1,4_]Cl. The Nyquist plots were best fitted to the equivalent circuit displayed in Figure 5. The values of the parameters obtained from the fitted data are listed in Table 1. Where, *R*_S_ is the solution resistance, *Q* is the constant phase element (CPE), *R*_P1_ is the polarization resistance between the cast iron surface and a layer formed during the immersion of the iron electrode in AG-seawater with and without ILs inhibitors for 1 h before the EIS measurements, *C*_dl_ is the double layer capacitance, and *R*_P2_ is the corrosion resistance between the formed surface layer/solution interface.

**Table 1:** Electrochemical impedance spectroscopy (EIS) parameters for the cast iron electrode in AG-seawater with and without ionic liquid (IL) inhibitors.

Sample	EIS Parameters					
	*R*_S_/Ω·cm^2^	*Q*		*R*_P1_/Ω·cm^2^	*C*_dl_/F·cm^−^^2^	*R*_P2_/Ω·cm^2^
		*Y*_Q_/F·cm^−^^2^	*n*			
AG-seawater	5.310	0.000948	0.73	2.340	0.000623	950
+1 mM [EMIm]Cl	6.043	0.000731	0.77	3.864	0.000531	1142
+5 mM [EMIm]Cl	7.471	0.000601	0.75	5.841	0.000332	1376
+1 mM [Py_1,4_]Cl	6.71	0.000643	0.45	5.072	0.000493	1212
+5 mM [Py_1,4_]Cl	8.245	0.000531	0.44	7.247	0.000382	1364

As shown in Figure 4, the Nyquist plots exhibit incomplete capacitive semicircles. The bigger the diameter of the semicircle usually means higher corrosion resistance. It is clear that the diameter increases up on the addition of the ionic liquids and with the increase of their concentrations signifying their inhibiting effect on the corrosion of cast iron in AG-seawater. This indicates that the corrosion inhibition influence enhances by the presence of the ILs and as their content increases. It is also seen from Table 1 that the *Q* with their *n* values greater than 0.5 and less than 1.0 for [EMIm]Cl represent double layer capacitors. On the other hand, the values for *Q* with their *n* values close to 0.5 in the case of [Py_1,4_]Cl account for a Warburg impedance. It is noticed that the presence of IL inhibitors, and also the increase of their concentrations, leads to a pronounced decrease in the CPEs values due to the increased passivity for the cast iron surface. This effect also leads to increasing the values of solution and polarization resistances (*R*_S_, *R*_P1_ and *R*_P2_). In addition, the values of *C*_dl_ lessen on addition of the employed ionic liquids, which is attributable to the increase in the thickness of the double layer owing to the adsorption of the ionic liquid species on the electrode surface.

**Figure 6:**
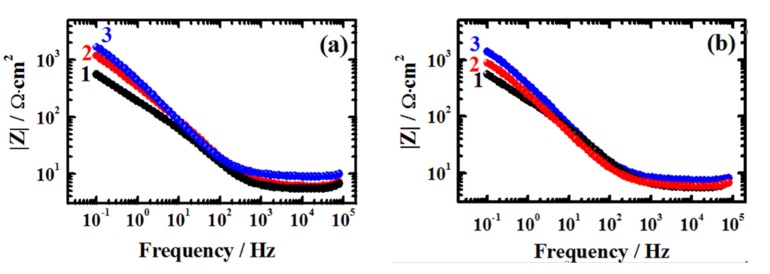
Bode impedance plots for the cast iron in AG-seawater without (1) and with (2) 1.0 mM and (3) 5.0 mM of (**a**) [EMIm]Cl and (**b**) [Py_1,4_]Cl.

The EIS results obtained from the Nyquist plots shown in Figure 4 were further enhanced by the Bode impedance of the interface plots shown in Figure 6 for the cast iron electrode in AG-seawater in the absence and the presence of the ILs inhibitors. It is seen from the plots of Figure 6 that the lowest impedance |*Z*| values over the whole frequency range were recorded for the cast iron immersed in the seawater in the absence of inhibitor. The presence of ILs inhibitors increased the absolute value of |*Z*| with change of the frequency, particularly at the low frequency area. This effect was also found to increase with the increase of ILs concentrations. According to the previous work [26], the increase of impedance of the interface at the low frequency area results from the increased passivity of the surface and in this case the passivation of the surface is due to the presence of ILs inhibitors and their adsorption on the cast iron surface protecting it from being corroded easily.

### 2.4. Cyclic Potentiodynamic Polarization

**Figure 7:**
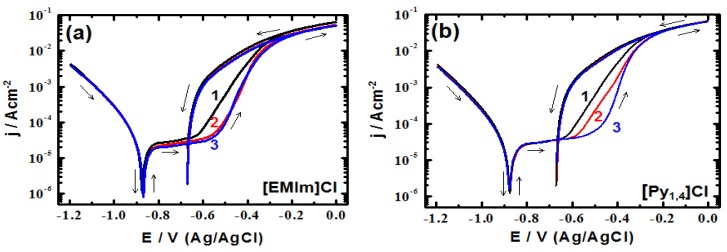
Cyclic potentiodynamic polarization for the cast iron in AG-seawater without (1) and with (2) 1.0 mM and (3) 5.0 mM of [EMIm]Cl (**a**) and [Py_1,4_]Cl (**b**).

Cyclic potentiodynamic polarization (CPP) experiments were also performed in the employed electrolyte in absence and presence of the ionic liquid inhibitors. The obtained corrosion parameters such as, corrosion potential (*E*_Corr_), pitting potential (*E*_Pit_) and protection potential (*E*_Prot_) are listed in Table 2. Figure 7 shows the CPP curves obtained for the cast iron electrode in AG-seawater without and with addition of the employed ionic liquids. The CPP curves were recorded after 1 h immersion in the test electrolyte. As shown in Figure 7, in the blank electrolyte without addition of ionic liquids, the cast iron electrode exhibits an active-passive behavior. A passive region of about 200 mV is observed on the anodic branch of the polarization curve as a result of the formation of an oxide film on the electrode surface. At the end of the passive region (*i.e.*, the pitting potential) the current increases rapidly with the potential scan, indicating the starting of passivity breakdown. In the backward scan a hysteresis loop is observed revealing the occurrence of pitting corrosion. On addition of [EMIm]Cl, Figure 7a, no significant change in the cathodic branch of the polarization curve is observed. However in the anodic branch, the passive region is significantly increased indicating the inhibiting influence of [EMIm]Cl on the corrosion of cast iron. The CPP curves obtained in the presence of [Py_1,4_]Cl, Figure 7b, show almost the same behaviour as the passive region increases and the pitting potential shifts to less negative values with the increase in the IL concentration. The corrosion potential (*E*_Corr_) does not show a significant change upon addition of the ionic liquid inhibitors, where as the protection potential (*E*_prot_) slightly shifts towards less negative values, Table 2.

The results of the CPP are in agreement with those of EIS and both are consistent with the weight loss results. The EIS and CPP results shown in Table 1 and Table 2, respectively, reveal a relatively higher inhibiting impact of [Py_1,4_]Cl compared with [EMIm]Cl. The nature and the strength of the adsorption of the employed ionic liquids on the electrode surface determine their inhibition efficiency. As stated above, the cations of the employed ionic liquids can be electrostatically adsorbed on the surface forming a corrosion barrier. As the charge of the imidazolium cation is delocalized, the adsorption might occur through the imidazoline ring, and hence the ring would be horizontally sited on the surface. However, in the case of [Py_1,4_]Cl the charge is localized thus, a strong interaction with the surface is obtained; the pyrrolidine ring would be perpendicularly positioned on the electrode surface.

**Table 2:** Polarization parameters obtained for the cast iron electrode in AG-seawater with and without IL inhibitors.

Electrolyte	CPP Parameters		
	*E*_Corr_/mV	*E*_Pit_/mV	*E*_Prot_/mV
AG-seawater	−878	−625	−668
+1 mM [EMIm]Cl	−875	−560	−664
+5 mM [EMIm]Cl	−872	−550	−662
+1 mM [Py_1,4_]Cl	−873	−570	−660
+5 mM [Py_1,4_]Cl	−876	−540	−655

## 3. Experimental Setup

### 3.1. Chemicals and Materials

The ionic liquids 1-ethyl-3-methylimidazolium chloride and 1-butyl-1-methylpyrrolidinium chloride with 99% purity and were purchased from Ionic Liquids Technologies GmbH (Heilbronn, Germany) and having the structure formulas shown in Scheme 1 were used as received. The AG-seawater was brought directly from the Arabian Gulf at the eastern region of Saudi Arabia. The chemical composition (in wt %) of the cast iron that was employed in the present study was as following; 4.55% C, 2.16% Si, 0.29% Mn, 0.083 P, 0.069 Mg, 0.050% Ti, 0.027% W, 0.022% Cr, and the balance was Fe.

### 3.2. Weight-Loss Measurements

The weight loss data were collected from rectangular cast iron coupons with dimensions of 40 mm length, 20 mm width, and 4 mm thickness. The coupons to be exposed to the solution were ground successively with metallographic emery paper of increasing fineness of up to 800 grits. Then, the coupons were washed with distilled water, degreased with acetone, washed using distilled water, and dried with tissue paper, respectively. The coupons were then weighed before being suspended in 300 cm^3^ solutions of AG-seawater without and with [EMIm]Cl and [Py_1,4_]Cl for different exposure periods (2–10 days). Each experiment was repeated three times for confirming the reproducibility of the test and the average loss in weight was taken. The losses in weight per area (Δm, mg·cm^−2^) and the corrosion rates (*R*_Corr_, mills per year (mpy)) over the exposure time were calculated as previously reported [26,27]:

(3) $$\Delta m\;=\frac{m_{1}-m_{2}}{A}$$

(4) $$R_{\text{Corr}}\;=\frac{543\Delta m}{Dt}$$

Here, *m*_1_ represents the weight of the cast iron coupon per mg before immersion in the AG-seawater, *m*_2_ representing the weight after are the weights of the cast iron coupon per mg after immersion, *A* refers to the area in cm^2^ (*A* = 20.8) for the cast iron coupon, *D* is the density (*D* = 7.563 g/cm^3^) for the cast iron coupon, and *t* is the time of exposure (h) of the cast iron coupon in the test solution. The percentage of inhibition efficiency (IE %) for [EMIm]Cl and [Py_1,4_]Cl on the corrosion of cast iron was also calculated as follows [28]:

(5) $$\text{IE \% }=\frac{R_{Corr}-R_{Corr}^{o}}{R_{Corr}}$$

where, *R*_Corr_ represents the corrosion rate of cast iron in the absence of inhibitor and *R*^o^_Corr_ is the corrosion rate in the presence of [EMIm]Cl and [Py_1,4_]Cl corrosion inhibitors.

### 3.3. Electrochemical Measurements

The electrochemical measurements were collected using a conventional three-electrode configuration electrochemical cell that only accommodates an amount of 200 cm^3^ of the test solution. The cast iron electrode, platinum sheet electrode, and an Ag/AgCl electrode (in 3.0 M KCl) were used as the working, counter, and reference electrodes, respectively. The working electrode had a square shape with dimensions of 1.0 cm length, 1.0 cm width and 3.0 cm height and total exposed surface area for the cast iron electrode in the seawater was exactly 1 cm^2^. Where, the cast iron electrode was prepared as reported in our previous work [27].

The electrochemical measurements were performed using an Autolab Potentiostat-Galvanostat (PGSTAT20) that was purchased from Metrohm Autolab B.V. (Utrecht, The Netherlands). The change of the open-circuit potential (OCP) was measured over the exposure time for 24 h at room temperature. Electrochemical impedance spectroscopy (EIS) experiments were obtained at corrosion potentials (*E*_Corr_) over a scanned frequency range of 100,000 Hz to 0.1 Hz, ± 5 mV peak-to-peak overlaid ac wave, and on a dc bias potential. The EIS results were acquired using the Powersine software by changing the frequency with a rate of 10 points per decade. For cyclic potentiodynamic polarization (CPP) measurements, the potential was scanned in the forward direction from −1.20 V to 0 V *vs.* Ag/AgCl using a scan rate of 0.001 V/s. After reaching the value of 0 V, the potential was directly rescanned in the reverse direction with the scan rate until the backward current intersects with the forward one. Each experiment was performed on a fresh surface of the cast iron electrode using a new portion of the electrolytic Arabian Gulf seawater.

## 4. Conclusions

The inhibiting influence of the ionic liquids [EMIm]Cl and [Py_1,4_]Cl on the corrosion of cast iron in AG-seawater was investigated. It was shown that the addition of the employed ionic liquids significantly decreases the corrosion of cast iron in AG-seawater. The free corrosion potential shifts to less negative values as the IL concentration increases and the extent of the potential shift is higher in case of [Py_1,4_]Cl. The weight loss results revealed that [EMIm]Cl exhibits a higher inhibition efficiency at short term immersion, whereas at long term immersion [Py_1,4_]Cl shows a higher efficiency. The cyclic potentiodynamic polarization and impedance spectroscopy results also demonstrated the corrosion inhibition impact of the employed ionic liquids and the results are consistent with those obtained from weight loss. The corrosion inhibition of the employed ionic liquids is ascribed to the adsorption of the cations onto the surface of cast iron forming a barrier between the surface and the aggressive electrolyte.
